# The Impact of a Tongue Training Device on Tongue Muscle Strength in Patients with Obstructive Sleep Apnea After Modified Uvulopalatopharyngoplasty: A Pilot Study

**DOI:** 10.3390/medicina61030511

**Published:** 2025-03-16

**Authors:** Yung-An Tsou, Chien-Hao Huang, Yu-Jen Chou, Hsueh-Hsin Kao, Jui-Kun Chiang, Yee-Hsin Kao

**Affiliations:** 1 Department of Otorhinolaryngology-Head and Neck Surgery, China Medical University Hospital, Taichung 404, Taiwan; d22052121@gmail.com (Y.-A.T.); j5287928@hotmail.com.tw (C.-H.H.); 2 Department of Otolaryngology Head and Neck Surgery, Asia University Hospital, Taichung 40402, Taiwan; 3 School of Medicine, China Medical University, Taichung 40402, Taiwan; 4 Department of Audiology and Speech-Language Pathology, Asia University, Taichung 41354, Taiwan; 5 Nature Dental Clinic, Nantou 545, Taiwan; webbchou1220@gmail.com; 6 Department of Radiation Oncology, Taichung Veterans General Hospital, Taichung 407, Taiwan; kaogrady8176@gmail.com; 7 Department of Family Medicine, Dalin Tzu Chi Hospital, Buddhist Tzu Chi Medical Foundation, No. 2, Minsheng Road, Dalin, Chiayi 622, Taiwan; 8 Department of Family Medicine, Tainan Municipal Hospital (Managed by Show Chwan Medical Care Corporation), 670 Chung-Te Road, Tainan 701, Taiwan

**Keywords:** Iowa Oral Performance Instrument (IOPI), tongue, strength, obstructive sleep apnea (OSA)

## Abstract

*Background and Objectives*: Sufficient evidence supports the Iowa Oral Performance Instrument (IOPI) as a reliable tool for measuring tongue strength and endurance as well as an effective assessment instrument for intervention studies. This study aimed to investigate the impact of a tongue training device, the HEAL, on tongue muscle strength in patients with obstructive sleep apnea (OSA) following modified uvulopalatopharyngoplasty (UPPP), utilizing the IOPI for evaluation. *Materials and Methods*: We introduced a tongue strengthener, the HEAL, made from medical-grade silicone, designed to improve tongue strength. Each patient was provided with the HEAL and instructed to begin using it one month after undergoing modified UPPP for OSA. The tongue strength of OSA patients was measured using the IOPI both before using the HEAL and approximately 6 weeks later. *Results*: A total of 66 participants with OSA underwent modified UPPP and were included in the final analysis. The mean age was 39.15 ± 8.84 years, and the mean BMI was 27.41 ± 4.03 kg/m^2^. The duration between the pre- and post-assessment of tongue strength using the HEAL was 42.95 ± 17.97 days. The mean tongue strength pressure before and after using the HEAL was 32.16 ± 16.00 kilopascals (kPa) and 42.95 ± 17.97 kPa, respectively. Tongue strength increased by an average of approximately 52.8% after using the HEAL for an approximate duration of 6 weeks. *Conclusions*: In the current study, participants with OSA who had undergone modified UPPP and subsequently used the HEAL demonstrated an average improvement of 10.79 kPa in tongue strength—an increase of over 50%—as measured by the IOPI. The interval between the pre- and post-assessment of tongue strength was approximately six weeks. Further studies are needed to validate these findings.

## 1. Introduction

Tongue strength training has been demonstrated to enhance swallowing pressure in patients with dysphagia, including those with cerebrovascular accidents [1,2], acquired head injuries [3], or Parkinson’s disease [4]. Tongue muscle strength is essential for various oral functions, such as food processing, taste and texture perception, and minimizing the risk of aspiration. Notably, improved tongue muscle strength may also offer additional benefits beyond these primary functions.

Orofacial myofunctional therapy (OMT) is designed to train and rehabilitate the muscles of the orofacial complex, including the tongue muscles, which control tongue positioning and gross movements; the lips and cheeks, which aid in lip sealing, articulation, cheek tension, and food manipulation during chewing; the jaw muscles, which facilitate jaw movement and chewing; the soft palate muscles, which elevate and tense the soft palate to assist with swallowing; the pharyngeal muscles, which support swallowing and airway function; and the hyoid and suprahyoid muscles, which play a role in swallowing, tongue movement, and stabilizing the hyoid bone.

A previous systematic review reported that OMT is effective in reducing the severity of obstructive sleep apnea (OSA) and snoring in adults [5]. A previous systematic review reported that individuals with OSA have reduced tongue elevation strength and endurance compared to healthy adults. However, this review highlighted the limited data on the effects of myofunctional therapy on tongue motor skills in OSA patients [6]. The mechanism may involve the genioglossus muscle, which plays a critical role in tongue protrusion. The activation of this muscle can help prevent airway collapse during sleep in patients with OSA [7]. Nevertheless, a randomized controlled trial found that six weeks of tongue elevation muscle training did not significantly reduce OSA severity [8]. Therefore, the impact of tongue elevation muscle training on OSA requires further investigation.

Continuous positive airway pressure is the standard first-line treatment for patients with OSA [9]. However, compliance rates vary widely, with 17% to 85% of patients failing to adhere to therapy, leaving many individuals untreated [10]. Consequently, alternative treatments, such as OMT targeting the upper airway muscles, have been investigated [5].

A previous systematic review and meta-analysis reported that the Iowa Oral Performance Instrument (IOPI) is a reliable tool for measuring tongue strength and endurance, as well as an effective assessment instrument for intervention studies [11]. We have introduced a tongue-strengthening device called the HEAL to improve tongue strength. The aim of this study is to evaluate the effectiveness of a tongue strengthener, the HEAL, in enhancing tongue strength in patients with OSA who have undergone modified UPPP. Tongue strength will be assessed using the IOPI.

## 2. Materials and Methods

### 2.1. Study Design

This observational study was conducted to examine the impact of the HEAL on tongue strength in patients with OSA following modified UPPP using the IOPI for assessment. The study protocol was reviewed and approved by the institutional review board of the Research Ethics Committee of the China Medical University Hospital, Taiwan (No. CMUH 110-REC3-200).

### 2.2. Study Population

A total of 76 patients with OSA sought treatment at the ENT department of a tertiary hospital in Taiwan between 7 March 2022 and 27 February 2023. Written informed consent was obtained from all participants prior to enrollment. In the current study, the inclusion criteria were as follows: adults aged 20 years or older who had been diagnosed with OSA and were scheduled to undergo a modified UPPP. The exclusion criteria were as follows: individuals who lacked the ability to act autonomously, those without teeth, and those with abnormal mental consciousness.

### 2.3. Interventions

**Figure 1:**
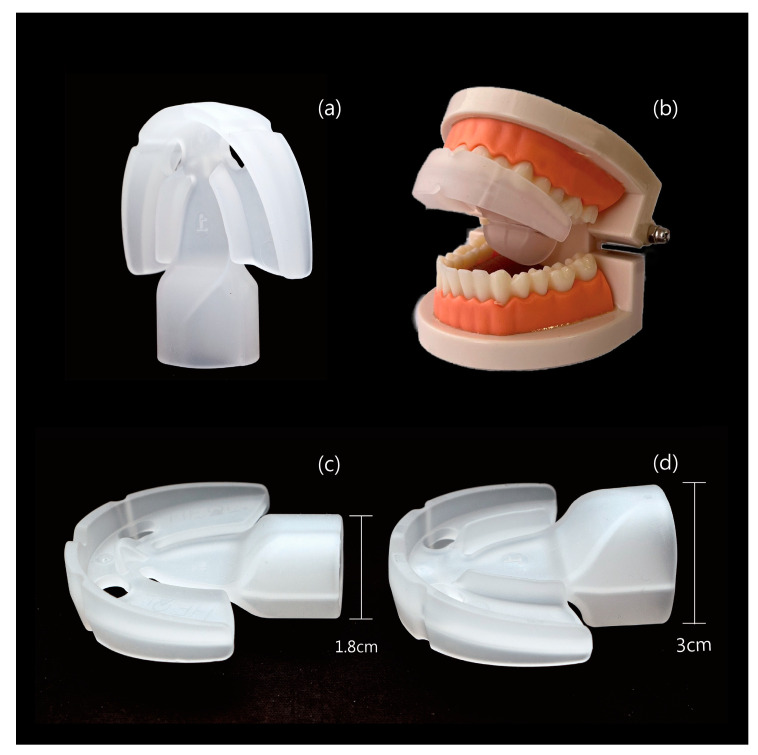
The structure of the novel tongue strengthener, the HEAL (Sinyuan Health Enterprise Co., Ltd., Taichung, Taiwan). (**a**) The vertical version. (**b**) The version positioned behind the dental mold. (**c**) The basic version, measuring 5.5 × 6.0 × 1.8 cm and featuring an upgraded tongue compressor. (**d**) The enhanced version, measuring 5.5 × 6.0 × 3.0 cm.

**Figure 2:**
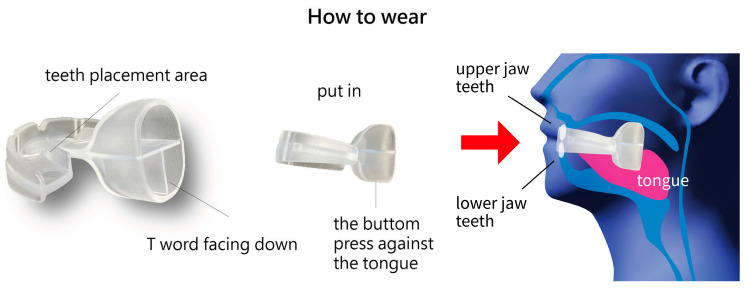
The procedure for using the HEAL.

We introduced a tongue strengthener, the HEAL, made from medical-grade silicone, designed to improve tongue strength. Each patient was provided with the HEAL (Patent No. M587995, Taiwan) [12] (Figure 1). The participants were instructed to start using the HEAL one month after undergoing modified UPPP for OSA. It is recommended to begin using the HEAL at least one month after modified UPPP to avoid the risk of wound bleeding. The device should be used during waking hours and is not recommended for use while sleeping. Each session should last 5–10 min, with a total daily training time of at least 30–40 min. The procedure for using the HEAL is shown in Figure 2. In the current study, tongue strength was assessed both before using the HEAL and 6 weeks afterward. Additionally, we conducted follow-ups every two weeks via telephone to monitor the condition of patients using the HEAL.

The HEAL is crafted from medical-grade silicone. The HEAL’s structure comprises several components, including a semi-circular main body with curved edges designed to fit the dental arch. The center features openings that facilitate airflow, improve stabilization, and reduce weight. Additionally, the rear protruding section, which is square and tubular, serves as a key component. It functions as a passive tongue compressor while also allowing active tongue pressing, thereby enhancing the effectiveness of tongue exercises. The HEAL is available in two versions: the basic version, measuring 5.5 × 6.0 × 1.8 cm, and the enhanced version, measuring 5.5 × 6.0 × 3.0 cm, featuring an upgraded tongue compressor. The HEAL strengthens the tongue through targeted exercises facilitated by the device. It employs both active and passive training mechanisms: active training involves the tongue pressing upward against the training structure and moving in circular motions along it, while passive training occurs as increased saliva production during wear triggers natural swallowing, causing the tongue to move autonomously and stimulate the training structure.

Tongue strength was evaluated using the Iowa Oral Performance Instrument (Model 2.2; IOPI Medical LLC, Carnation, WA, USA), which consists of a plastic catheter and a balloon. During the measurement, participants were instructed to sit in a relaxed position and apply maximum pressure to the balloon by squeezing it for 3 s. Tongue strength was assessed specifically during tongue elevation. Measurements were taken before the start of the tongue strength training with the HEAL and again after six weeks, with each assessment repeated three times. We assessed tongue strength in the posterior part of the tongue.

In this study, we used the Berlin Questionnaire (BQ) to assess OSA, as it is a useful screening tool for the general population due to its good sensitivity and high negative predictive value in ruling out severe OSA [13]. The BQ assigns scores across three categories, with a score of 2 or more in at least two categories indicating a high likelihood of sleep apnea. The 10-item Eating Assessment Tool (EAT-10) was used in the present study to evaluate swallowing function. It has demonstrated excellent internal consistency, test–retest reliability, and criterion-based validity. Normative data indicate that an EAT-10 score of 3 or higher is considered abnormal [14].

### 2.4. Outcome Measures

The objective of this study is to assess and compare tongue strength before and after 6 weeks of using the HEAL in patients with OSA who have undergone UPPP using the IOPI for evaluation.

### 2.5. Statistical Analysis

The statistical analyses were conducted using the R software (version 4.4.1; R Foundation for Statistical Computing, Vienna, Austria). The statistical significance was set at *p* < 0.05, and all tests were two-tailed. The categorical variables are presented as frequencies and percentages, while the continuous variables are expressed as means ± standard deviations as well as a mean ± standard error in bar plot figures. The *t*-test or Wilcoxon rank-sum test was used to compare continuous variables between two groups (male and female). A paired *t*-test was applied for the comparison of the dependent data. Fisher’s exact test was used for the count data. A scatter plot was drawn to visualize the data. Loess regression is the method used to smoothen a time series to plot the difference of pre- and post-tests. A linear regression analysis was performed to identify significant factors affecting the outcome.

## 3. Results

A total of 76 participants with OSA sought assistance at the ENT department. After excluding 10 participants, 66 (86.8%) who underwent modified UPPP for OSA were enrolled in the current study for analysis. The mean age was 39.15 ± 8.84 years, and the mean BMI was 27.41 ± 4.03 kg/m^2^. A total of nine patients (13.6%) had a history of hypertension, two (3%) had diabetes, thirteen (19.7%) were smokers, and six (9.1%) consumed alcohol. The duration between the pre- and post-assessments of tongue strength using the HEAL was 42.95 ± 17.97 days. We assessed tongue strength in the posterior part of the tongue. Before using the HEAL, the mean tongue strength pressure (mean ± S.D.) was 32.16 ± 16.00 kPa, which increased to 42.95 ± 17.97 kPa after use. This represents an average improvement of 10.79 kPa in tongue strength pressure. Tongue strength increased by approximately 52.8% [(49.15 − 32.16)/32.16] after using the HEAL. There were no significant differences between males and females (Table 1).

The relationship between the pre- and post-assessments of HEAL usage was analyzed using univariate linear regression, showing no significant differences in the variables listed in Table 1 (Table 2). Similarly, no significant differences were found in the multivariate linear regression analysis.

**Figure 3:**
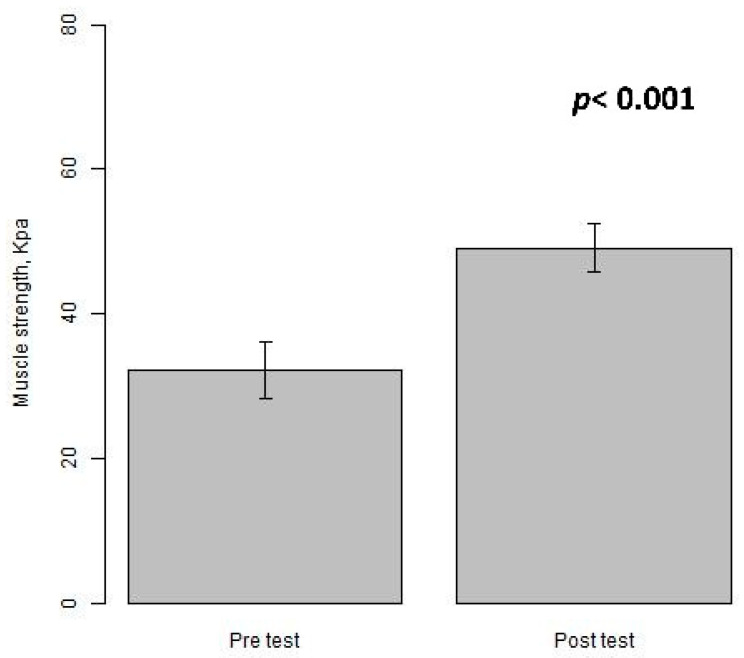
The bar plots (mean ± S.E.) compare tongue muscle strength between pre- and post-assessment measurements using IOPI while using the HEAL.

**Figure 4:**
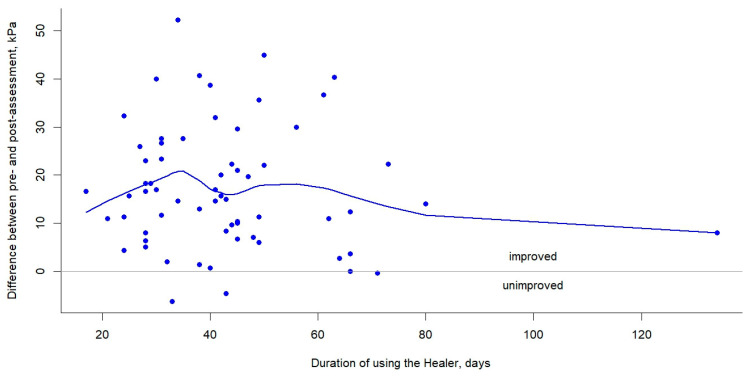
The scatter plots with a smoothing curve illustrate the variation in the difference between pre- and post-assessment tongue strength over time with the use of the HEAL.

The bar plots show improved tongue muscle strength (measured by IOPI) after using the tongue-strengthening device (HEAL) for an approximate duration of 6 weeks (*p* < 0.001) (Figure 3). The scatter plots with a smooth line illustrate the changes in tongue strength for the pre- and post-assessments over time while using the HEAL, with the majority of participants (62/66 = 93.9%) demonstrating improvement (Figure 4). 

## 4. Discussion

The tongue-strengthening device, the HEAL, introduced in this study, is a non-invasive tool designed to facilitate isometric exercises for tongue strength rehabilitation. Participants with OSA who had undergone modified UPPP—primarily transoral robotic surgery with UPPP—and then traditional exercises combined with the use of the HEAL showed an improvement of over 50% in tongue strength, as measured by the IOPI. The difference in mean tongue strength pressure before and after using the HEAL indicates an improvement of 10.79 kPa on average. The average duration between pre- and post-assessment was approximately six weeks.

Traditional oral strength therapies encompass a variety of established techniques, including oromotor sensory stimulation [15], the Mendelsohn maneuver [16], supraglottic swallowing [17], range-of-motion exercises, oromotor strengthening [18], chin-down/chin-tuck positioning [19], head-turning toward the weaker side, tilting toward the stronger side [20], effortful swallowing [21], and cough training [22]. Physiatrists and occupational therapists carefully select specific exercises, maneuvers, or dietary modifications to address the unique needs and impairments of each patient.

A systematic review reported significantly higher anterior tongue strength and posterior tongue strength in the tongue-strengthening exercise intervention compared to the control group, with increases of 5.34 kPa and 8.12 kPa, respectively [23]. A previous study reported improvements in tongue strength following an isometric lingual exercise program in stroke patients with dysphagia. After four and eight weeks of exercise, tongue strength increased by 27.2% and 56.0%, respectively, in the anterior tongue and by 45.5% and 80.8%, respectively, in the posterior tongue. The exercise involved compressing an air-filled bulb between the tongue and the hard palate [2]. Another previous study reported that after four weeks of tongue strength training using the Iowa Oral Performance Instrument in stroke patients with dysphagia, tongue strength increased by 9.5% in the anterior tongue and 14.0% in the posterior tongue [24]. A previous study reported that after six weeks of traditional exercises combined with an isometric lingual resistance exercise program—incorporating active resistance in all directions (protrusion, lateralization, and elevation) using the tongue against a tongue depressor—oral strength improved by 4.2% in patients with oral and oropharyngeal cancer who had been treated with primary radiotherapy, with or without chemotherapy [25]. A previous study reported that tongue strength decreases with age and is generally greater in males than in females. Individuals aged 79 years and older exhibited a statistically significant decline in tongue strength [26]. Compared to previous studies, the use of the HEAL resulted in an average improvement of 10.79 kPa in tongue strength, which represents an increase of over 50% after an average duration of approximately six weeks between the pre- and post-assessments in the current study. This is similar to the findings of Robbins et al., who conducted an isometric lingual exercise program involving compressing an air-filled bulb between the tongue and the hard palate, with improvements of 45.5% and 80.8% after four and eight weeks of exercise, respectively, as measured using the IOPI [2]. However, the study populations were different: the current study involved individuals with OSA who had received modified UPPP, while Robbins et al.’s study focused on stroke patients with dysphagia [2].

A common discomfort experienced during the early stages of using the HEAL is nausea, which may subside over time. To help alleviate this, it is recommended to perform tongue warm-up exercises before wearing the device.

The IOPI functions as both a tool for measuring tongue strength and a device for tongue-strengthening exercises. Its advanced features, including pressure-sensing circuitry, a peak hold function, and a timer, make it highly effective for biofeedback-based training. Patients can perform isometric tongue exercises with the IOPI by pressing its air-filled bulb against the palate, gradually improving tongue strength. This dual-purpose design makes the IOPI a versatile instrument for both assessment and therapeutic applications in oral strength training [11]. However, its high cost limits accessibility for home use, and it requires multiple connective components and air-filled bulbs that are susceptible to leaks. Additionally, the material properties of the device may degrade over time due to use and deformation.

Continuous positive airway pressure (CPAP) therapy is currently the first-line treatment for OSA in adults [27]. However, due to low compliance with CPAP, oral appliances are recommended as an alternative for individuals who cannot tolerate CPAP or prefer other options [28]. Oral appliances include mandibular advancement devices, mandibular repositioning devices, mandibular advancement splints, mandibular advancement appliances, and others. Surgical treatments for OSA aim to enhance airway patency by addressing specific sites of obstruction. These surgical options include extended uvulopalatoplasty [29], multilevel (tongue and palate) temperature-controlled radiofrequency tissue ablation [30], and uvulopalatopharyngoplasty (UPPP) [31].

The human tongue consists of two main types of muscle fibers. The posterior tongue is composed primarily of type I slow-twitch muscle fibers, which are responsible for sustained tonic activities, such as maintaining retroglossal airway patency [32,33]. In contrast, the anterior tongue contains type II fast-twitch muscle fibers, which are responsible for short bursts of activity, such as chewing and speaking [34]. We assessed only posterior tongue strength because the patients enrolled in this study had OSA and had undergone modified UPPP, which was a limitation of this study.

Many oral strength and swallowing devices are available on the market. We introduce an oral strength device called the HEAL, specifically designed to improve tongue strength. The key advantage of the HEAL lies in its rear protruding section, which functions as a passive tongue compressor while also enabling active tongue pressing, thereby enhancing the effectiveness of tongue exercises. Moreover, the rear protruding section is available in two versions: the basic version (5.5 × 6.0 × 1.8 cm) and the enhanced version (5.5 × 6.0 × 3.0 cm), which features an upgraded tongue compressor for improved performance.

There are several limitations in the current study. Firstly, the sample size was relatively small; only 66 participants (including 53 males and 13 females) were enrolled for the analysis. Secondly, there were missing data for ten patients (13.2%), possibly due to the lack of an assessment before HEAL use or irregular follow-up. Further research is needed to establish a detailed training protocol for using the HEAL. Thirdly, the detraining effects were not assessed. The detraining effect occurs when patients stop tongue-strengthening exercises, leading to a decline in tongue muscle strength. Fourthly, we assessed tongue strength only in the posterior part of the tongue, not the anterior part. However, posterior tongue strength may be associated with OSA. Finally, we found that the HEAL can be combined with surgical treatments for OSA to enhance tongue muscle strength, particularly in the posterior tongue. However, the effects of HEAL on the further increase in tongue muscle strength and its impact on changes in OSA severity require investigations in future studies. Finally, the lack of a control group and the relatively short follow-up period (less than 4 months) represent limitations of the current study. Nevertheless, from the smoothing curve in Figure 4, it can be discerned that in the patients of this study, the changes in tongue muscle strength between the pre- and post-tests exhibit an initial increase, followed by a decline in the later stages, which corresponds with the duration of training in days.

## 5. Conclusions

The tongue-strengthening device, the HEAL, introduced in this study, is a non-invasive tool designed to facilitate isometric exercises for tongue strength rehabilitation. Participants with OSA who had undergone modified UPPP showed an improvement of 10.79 kPa in mean tongue strength pressure when combining traditional exercises with the use of the HEAL. This represents a greater than 50% increase in tongue strength, as measured by the IOPI. The average duration between the pre- and post-assessments was approximately six weeks. Further studies with a larger sample size and a prospective design are warranted to verify the results.

## Figures and Tables

**Table 1:** Demographics and characteristics of the enrolled participants.

Variables	Total	Female	Male	*p*
N	66	13	53	
Age, years old	39.15 ± 8.84	41.01 ± 7.50	38.69 ± 9.14	0.209
BMI, kg/m^2^	27.41 ± 4.03	27.05 ± 4.87	27.50 ± 3.84	0.722
Smoking history	13 (19.7%)	2 (15.4%)	11 (20.8%)	1
Alcohol history	6 (9.1%)	0	6 (11.3%)	0.589
Hypertension	9 (13.6%)	2 (15.4%)	7 (13.2%)	1
Diabetes	2 (3%)	1 (7.7%)	1 (1.9%)	0.358
EAT-10	5.15 ± 9.02	4.62 ± 8.93	5.28 ± 9.12	0.833
The score of EAT-10 ≥ 3	22 (33.3%)	3 (23.1%)	19 (35.8%)	0.518
The score of Berlin Questionnaire ≥ 2	45 (68.2%)	8 (61.5%)	37 (69.8%)	0.741
AHI, events/h	33.14 ± 28.2	16.24 ± 11.88	37.63 ± 29.63	0.023
IOPI before the HEAL use, kPa	32.16 ± 16.00	29.31 ± 13.78	32.86 ± 16.54	0.478
Duration of training, days	42.95 ± 17.97	39.31 ± 13.91	43.85 ± 18.83	0.5449
IOPI after the HEAL use, kPa	49.15 ± 13.20	49.41 ± 14.17	49.09 ± 13.09	0.938

Abbreviation: AHI, apnea−hypopnea index; BMI, body mass index; EAT-10, the 10-item Eating Assessment Tool; IOPI, Iowa Oral Performance Instrument.

**Table 2:** The relationship between pre- and post-assessments of the HEAL usage was analyzed using univariate linear regression.

Variables	Estimate	S.E.	t Value	*p*
Age, years old	−0.35	0.54	−0.66	0.514
Male	−3.87	3.93	−0.99	0.328
BMI, kg/m^2^	1.91	1.16	1.65	0.104
Smoking history	19.46	11.62	1.68	0.099
Alcohol history	−0.06	5.47	−0.01	0.992
Hypertension	1.55	4.58	0.34	0.736
Diabetes	−6.70	9.14	−0.73	0.466
EAT-10	−0.17	0.17	−0.98	0.330
The score of EAT-10 ≥ 3	0.871	3.34	0.26	0.795
The score of Berlin Questionnaire ≥ 2	2.67	3.36	0.79	0.430
AHI, events/h	0.06	0.06	1.14	0.258

Abbreviation: AHI, apnea−hypopnea index; BMI, body mass index; EAT-10, the 10-item Eating Assessment Tool; IOPI, Iowa Oral Performance Instrument.

## Data Availability

The data engendered during and/or analyzed in this current study are not publicly available but can be obtained by request from the corresponding author, Y.H.K.

## Abbreviations

The following abbreviations are used in this manuscript:

BQ	Berlin Questionnaire
CPAP	Continuous positive airway pressure
EAT-10	The 10-item Eating Assessment Tool
ENT	Ear, nose, and throat
IOPI	Iowa Oral Performance Instrument
OMT	Orofacial myofunctional therapy
OSA	Obstructive sleep apnea
UPPP	Uvulopalatopharyngoplasty
